# A fully automated approach for baby cry signal segmentation and boundary detection of expiratory and inspiratory episodes

**DOI:** 10.1121/1.5001491

**Published:** 2017-09-11

**Authors:** Lina Abou-Abbas, Chakib Tadj, Hesam Alaie Fersaie

**Affiliations:** Department of Electrical Engineering, École de Technologie Supérieure, Quebec University, 1100 Rue Notre Dame Ouest, Montréal, Quebec H3C 1K3, Canada

## Abstract

The detection of cry sounds is generally an important pre-processing step for various applications involving cry analysis such as diagnostic systems, electronic monitoring systems, emotion detection, and robotics for baby caregivers. Given its complexity, an automatic cry segmentation system is a rather challenging topic. In this paper, a framework for automatic cry sound segmentation for application in a cry-based diagnostic system has been proposed. The contribution of various additional time- and frequency-domain features to increase the robustness of a Gaussian mixture model/hidden Markov model (GMM/HMM)-based cry segmentation system in noisy environments is studied. A fully automated segmentation algorithm to extract cry sound components, namely, audible expiration and inspiration, is introduced and is grounded on two approaches: statistical analysis based on GMMs or HMMs classifiers and a post-processing method based on intensity, zero crossing rate, and fundamental frequency feature extraction. The main focus of this paper is to extend the systems developed in previous works to include a post-processing stage with a set of corrective and enhancing tools to improve the classification performance. This full approach allows to precisely determine the start and end points of the expiratory and inspiratory components of a cry signal, EXP and INSV, respectively, in any given sound signal. Experimental results have indicated the effectiveness of the proposed solution. EXP and INSV detection rates of approximately 94.29% and 92.16%, respectively, were achieved by applying a tenfold cross-validation technique to avoid over-fitting.

## I. INTRODUCTION

Cry signals have been the object of research and analysis for many years. Researchers have found sufficient evidence that cry signals can provide relevant information about the physical and psychological states of newborns (Barr, 2006; Farsaie Alaie *et al.*, 2016; Golub, 1979; Soltis, 2004). Over the years, a considerable number of works have been conducted in the field of infant cry analysis. Orozco-García and Reyes-García (2003), Várallyay (2006), Hariharan *et al.* (2012), and Reyes-Galaviz *et al.* (2008) described efficient classification algorithms for distinguishing cries of normal infants from those of hypoacoustic infants, and as a result accuracy rates ranging from 88% to 100% were obtained. The accuracy rates for classification tasks between healthy infants and infants with asphyxia, as performed by Sahak *et al.* (2010) and Zabidi *et al.* (2010) were reported to be 93.16% and 94%, respectively. Moreover, anger, pain, and fear detection from cry signals were carried out by Petroni *et al.* (1995), yielding a recognition rate of 90.4%.

A newborn cry-based diagnostic system (NCDS) aims to achieve preliminary screening of newborn pathologies by analyzing the features of audible cry components detected in a realistic clinical environment (Farsaie Alaie *et al.*, 2016).

According to the World Health Organization, “every year, approximately 40% of child deaths are deaths of newborn infants in their first 28 days of life, 75% of newborn deaths occur in the first week of life, and up to two-thirds of newborn deaths can be prevented if known.”

Therefore, any technique that can contribute in identifying the very first signs of newborn diseases could have a great influence on decreasing infant mortality. Specifically, this is the main goal of our project: to develop a fully automatic noninvasive system able to diagnose diseases based solely on cry sound analysis. The implementation of such a diagnostic system first addresses the issue of finding the useful cry components in an input waveform. The NCDS may suffer in terms of intelligibility if the input audio file contains acoustical activities other than crying. Therefore, one of the challenges in implementing such a system is to create an automatic segmentation system to correctly locate the expiratory and inspiratory phases of a cry sequence. Despite the significant amount of research conducted on pathological cry signal classification, little has been done to address the problem of automatic segmentation of audible expiratory and inspiratory phases of crying. If we could automatically segment and identify important parts of a given recorded signal, it would be easier to develop a fully automatic diagnostic system. Such a system could hopefully be used as a real-time clinical decision support tool, and in the case of early detection of a symptom, the necessary treatment could be provided easily and cheaply.

This paper is organized as follows. Motivation and literature review are presented in Sec. II. Section III describes the corpus. Section IV details our proposed solution and describes the post-processing stage. The results and discussion are presented in Sec. V and, finally, Sec. VI concludes the paper by summarizing the main contribution of this work and describing future work.

## II. LITERATURE REVIEW AND MOTIVATION

The main components of a cry sound are expiration and inspiration segments with vocalization and audible expiration and inspiration (EXP and INSV, respectively).

The main challenge of this work is to develop a method able to locate EXP and INSV correctly within a given audio signal.

The problem of cry segmentation/detection cannot be considered a problem of voiced/unvoiced detection because a single audible cry can typically contain both voiced and unvoiced segments.

Alternately, the problem of cry detection in a corpus recorded in a very noisy clinical environment cannot be solved simply by traditional voice activity detection (VAD) modules, which are common in previous cry analysis systems proposed in the literature (Kuo, 2010; Ruiz *et al.*, 2010; Várallyay, 2006; Zabidi *et al.*, 2009). VAD refers to the issue of locating speech regions from other acoustic activity regions in a given audio signal. The other acoustic activity regions can be any type such as noise, silence, or an alarm warning. However, the signal-to-noise ratio (SNR) represents a crucial parameter and may result in major errors. VAD is an essential part of various audio communication systems such as automatic speech recognition, mobile phones, personal digital assistants, and real-time speech transmission. Common VAD methods are composed of two main modules: (1) feature extraction and (2) decision rules. Common features are those that depend on energy calculation of the signal, cepstral coefficients, zero-crossing rate (ITU, 1996), spectrum analysis (Marzinzik and Kollmeier, 2002), entropy and wavelet transforms (Wang and Tasi, 2008; Juang *et al.*, 2009). The decision rule is often formulated on a frame-by-frame basis and simple thresholding rules.

We applied well-known VAD algorithms used in G.729b and the Rabiner-Sambur method to detect cry segments (Benyassine *et al.*, 1997; Rabiner and Sambur, 1975). We found that:

• Threshold settings were not easy to select in a variable and noisy environment.
• Traditional VAD could not distinguish between important cry segments (EXP and INSV) and speech segments recorded during the data acquisition.
• Traditional VAD failed in distinguishing expiration phases from inspiration phases of cry signals.

To solve the issue of adjusting thresholds, statistical approaches seem to be a good solution. That is why we have given due consideration in our recent and present works to statistical model-based approaches (Abou-Abbas *et al.*, 2015b; Abou-Abbas *et al.*, 2015c).

Compared to other audio-related fields, such as speech and music, investigation of cry segmentation has seen low consideration. We refer readers to the state of the art discussed in our recent works (Abou-Abbas *et al.*, 2015b; Abou-Abbas *et al.*, 2015a).

Thus far, existing cry segmentation algorithms have mainly been able to separate cries from silent pauses or respiratory phases (Várallyay *et al.*, 2008, 2009). Noisy backgrounds or other acoustic activities have not been considered in these works because the database used was collected in a laboratory environment and not in a real clinical environment. For example, in a recent work (Aucouturier *et al.*, 2011), authors applied an automatic segmentation approach based on a hidden Markov models (HMMs) classification system to segment the expiratory and inspiratory parts of cry signals. Due to the limited number of infants and limited available acoustic activities in the corpus existing, the authors considered three classes expiration, inspiration, and silence. To train their models, a support vector machine (SVM), as well as Gaussian mixture models (GMMs) classifiers were used, and considering the arrangement in time between the three classes, a second stage using the Viterbi algorithm was added to consider the whole architecture as HMM.

Cry segmentation or detection has also been studied in Kim *et al.* (2013) and Yamamoto *et al.* (2010, 2013). The work of Kim *et al.* (2013) investigated a new feature called segmental two-dimensional linear frequency cepstral coefficients (STDLFCCs), which is based on linear frequency cepstral coefficients (LFCCs). The idea behind it was to capture the lower frequency as well as the higher frequency within long-range crying segments and provide better discrimination between crying and non-crying segments. An average equal error rate of 4.42% was reported in their article.

In Yamamoto *et al.* (2010, 2013), a method for detecting a baby's voice using a well-known speech recognition system called JULIUS and fundamental frequency analysis was introduced achieving a detection rate of 69.4%.

## III. THEORETICAL BACKGROUND

Like any audio recognition/detection system, a cry segmentation system can be briefly analyzed using three main modules:

• Feature selection and extraction, where suitable information is estimated in a relevant form and size from a cry signal to represent it in a different and more convenient domain;
• Classification, where representative models are created and adapted using extracted feature vectors for each available pathology class;
• Decision-making.

### A. Overview on features extraction

Cry signals can be described by their features within two common domains: (1) the time domain and (2) frequency domain. From either of the mentioned domains, a number of significant characteristics can be extracted (Scherer, 1982). In this section, we describe the different domain features applied at different levels of our work.

#### 1. Time-domain features

##### a. Intensity.

The intensity is also called the loudness, and it is related to the amplitude of the signal. It represents the amount of energy a sound has per unit area. The intensity is defined by the logarithmic measure of a signal section of length *N* in decibels as follows:

$$I=10\;\;\text{log}\;\left(\sum_{n=1}^{N}s^{2}(n)w(n)\right),$$

where *w* is a window function and *s*(*n*) is the amplitude of the signal.

Intensity is an essential feature widely used in different applications, such as music mood detection (Lu *et al.*, 2006), and an accuracy rate of 99% is achieved, proving the good performance of intensity features.

**FIG. 1.**
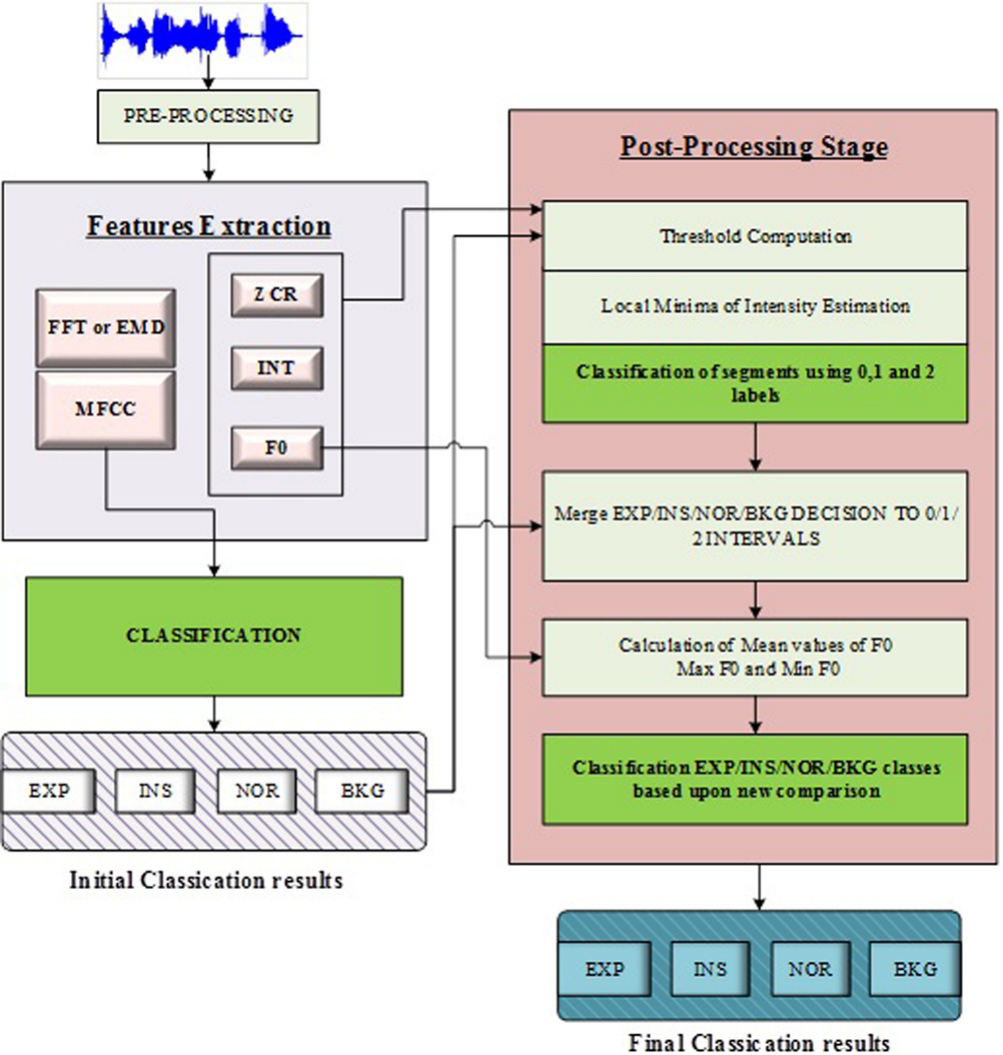
(Color online) Block diagram of the proposed approach combining the initial classification stage with the post-processing stage.

Considering Fig. 1, one can note that the intensity was increased considerably during the period of cry segments.

##### b. Zero crossing rate.

It is the one of the most widely used time-domain features in VAD algorithms (Bachu *et al.*, 2010; Rabiner and Sambur, 1975). Zero crossing occurs when consecutive samples have different algebraic signs as defined by Shete *et al.* (2014). Therefore, zero crossing rate (ZCR) represents the number of times in a specific frame the amplitude of the signal *x* (*n*) crosses the zero axis.

#### 2. Frequency-domain features

##### a. Fundamental frequency or pitch.

The time between successive vocal fold openings is called the fundamental period, *T*_0_, while the rate of vibration is called the fundamental frequency of the phonation, *F*_0_ = l/*T*_0_. The term pitch is often used interchangeably with fundamental frequency. However, there is a subtle difference. Psycho-acousticians use the term pitch to refer to the perceived fundamental frequency of a sound, whether or not that sound is actually present in the waveform (Deller *et al.*, 1993).

**FIG. 2.**
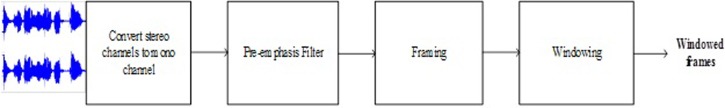
(Color online) Block diagram of the pre-processing stage.

It is one of the most used features in many applications, such as vowel classification, emotion classification, and music recognition. We have noticed that there are differences between expiratory and inspiratory phases regarding their frequency range (see Fig. 2).

There are several ways to estimate the fundamental frequency, for example, cross- and auto-correlation. In this work, we employed the classic auto-correlation method by using PRAAT software (Boersma and Van Heuven, 2001). Traditional statistical features, such as maximum, minimum, and mean of the fundamental frequency, were calculated over the entire corresponding utterance.

#### 3. Time-frequency features

##### a. Fast Fourier transform–based Mel frequency cepstrum coefficient.

Mel frequency cepstrum coefficients (MFCCs) are introduced in Davis and Mermelstein (1980) as the discrete cosine transform of the log-energy output of the triangular bandpass filters. MFCCs are used to represent the human speech signal by calculating the short term power spectrum of the acoustic signal based on the linear cosine transform of the log power spectrum on a nonlinear Mel scale of frequency. Mel scale frequencies are distributed in a linear space in the low frequencies (below 1000 Hz) and in a logarithmic space in the high frequencies (above 1000 Hz; Rabiner and Juang, 1993).

The steps from the original input signal to MFCCs are as follows:

• Split the signal into small overlapped frames of *N* samples;
• Decrease discontinuity between consecutive frames by employing the Hamming window defined as follows:
  $$w(n)=0.54-0.46\;\;\text{cos}\left(\frac{2\pi n}{N-1}\right),\;\;\;\;0\leq n\leq N-1\;;$$
• Apply the fast Fourier transform (FFT) and compute the power spectrum of the signal;
• Apply the log amplitude of the spectrum to the Mel scale filter banks where *f* denotes the real frequency
  $$\text{Mel}(f)=2595\times {\text{log}}_{10}\left(1+\frac{f}{700}\right)\;;$$
• Perform the inverse of the FFT, and the resulting amplitudes of the spectrum are MFCCs according to the equation
  $$c_{n}=\sum_{k=0}^{n-1}\;\text{log}(S_{k})\text{cos}\left[n\left(k-\frac{1}{2}\right)\frac{\pi }{k}\right],\;\;\;\;n=1,2,\ldots ,K,$$
  where $S_{k}$ is the output power spectrum of filters and *K* is chosen to be 12.

##### b. Empirical mode decomposition-based MFCC.

Empirical mode decomposition (EMD) is used for analyzing nonlinear and nonstationary signals and was proposed by Huang *et al.* (1998). EMD breaks down a given signal into intrinsic mode functions (IMFs) and a residual function during a sifting process. The main advantage of EMD is that basic functions are obtained directly from the signal. An IMF represents a simple oscillatory mode of the signal and is defined to draw the position of the signal in time. Each IMF must fulfill the following two basic criteria (Huang *et al.*, 1998):

• The number of ZCRs and extremes in the entire sequence of data must be equal or differ by only one.
• The mean value of the envelope defined by the local maxima and the envelope defined by local minima must be zero at any point.
• To extract an IMF from a given signal, the following steps are necessary (Huang *et al.*, 1998):
• Identify the extrema of the signal x(t) separately.
• Using the cubic splines interpolation method, interpolate the local maxima to form the upper envelope u(t).
• Using the cubic splines interpolation method, interpolate the local minima to form the lower envelope l(t).
• Consider the local mean value of the upper and lower envelopes m (t)=[u(t)+l(t)]/2.
• Consider the local mean value from the original signal h(t)=x(t)−m(t).
• Repeat the sifting approach until *h*(*t*) satisfies the basic criteria to be an IMF.
• Finally, the residue r(t)=x(t)−h(t) is regarded as the new signal to repeat the sifting process for the extraction of the second IMF, and so on.

The EMD-based MFCC features are calculated by applying the MFCC extraction technique to the IMFs instead of the original signal. Based on the results obtained from our previous works (Abou-Abbas *et al.*, 2017; Abou-Abbas *et al.*, 2015c) regarding finding the best IMF combination, we have found that the parameters extracted from the sum of IMF3, IMF4, and IMF5 yielded the best cry segmentation results. Therefore, this combination was employed in this work: IMF345 = IMF3 + IMF4 + IMF5.

#### 4. Differential and acceleration coefficients

The features extracted from the decomposed frame describe only the static features of the corresponding segment. To add dynamic information and extract both linear and nonlinear properties, delta and delta-delta coefficients were used. The dynamic information obtained by delta and delta-delta can be merged with static information to form one feature vector.

### B. Overview on classification approaches

#### 1. GMM

GMMs are commonly used for different applications, mostly speech recognition, speaker verification, and emotion recognition systems (Reynolds and Rose, 1995). It can be represented as a weighted sum of Gaussian distributions as follows:

$$p(o|\lambda )=\sum_{j=1}^{J}w_{j}G(o:\mu_{j},\Sigma_{j}),$$

$$G(o;\mu_{j},\Sigma_{j})={\left(2\pi \right)}^{-(D/2)}{\left|\Sigma_{i}\right|}^{-(1/2)}\;\text{exp}\;\left\{-\frac{1}{2}{\left(o-M_{i}\right)}^{T}\Sigma_{i}^{-1}(o-M_{i})\right\},$$

where p(o|λ) is the likelihood of the input observation *o* with the dimensionality of *D*, *J* is the number of mixtures, $w_{j}$ are the weighting coefficients satisfying the constraint $\sum_{j=1}^{J}w_{j}=1$, and $G(o_{j},\mu_{j},\Sigma_{j})$ denotes the *j*th Gaussian with mean vector $\mu_{j}$ and covariance matrix $\Sigma_{j}$.

#### 2. HMM

HMM-based methods have many potential applications in Airborne Surveillance Radar (ASR) systems, statistical signal processing, and acoustic modeling, including the segmentation of recorded signals (Young *et al.*, 2002). The basic principles of any ASR system involve constructing and manipulating a series of statistical models that represent the various acoustic activities of the sounds to be recognized (Young *et al.*, 2002). Many studies have shown that speech, music, newborn cries, and other sounds can be represented as a sequence of feature vectors (temporal, spectral, or both), and HMMs could provide a very important and effective framework for building and implementing time-varying spectral vector sequences (Gales and Young, 2007).

An HMM generates a sequence of observations *O* = *O*_1_,*O*_2_,…,*O_T_* and is defined by the following parameters: the number of hidden states, state transition probability distribution *A*, observation probability distribution *B*, and initial state distribution π. We denote the model parameters of the HMM as λ = {*A*,*B*,π} (Kuo, 2010; Várallyay, 2006).

To build and manipulate an HMM, three problems must be solved: the evaluation problem, the decoding problem, and the training problem. HMM theory, the aforementioned problems, and proposed solutions are widely explained in the literature, especially in the well-known Rabiner tutorial (Rabiner, 1989). Moreover, the Viterbi algorithm was proposed as a decoding solution to find the most probable future state of the system based on its current state (Rabiner, 1989). The Baum Welch algorithm is an iterative procedure used to estimate the HMM parameters.

## IV. GENERAL ARCHITECTURE AND SHORTCOMINGS OF PRIOR WORKS

**FIG. 3.**
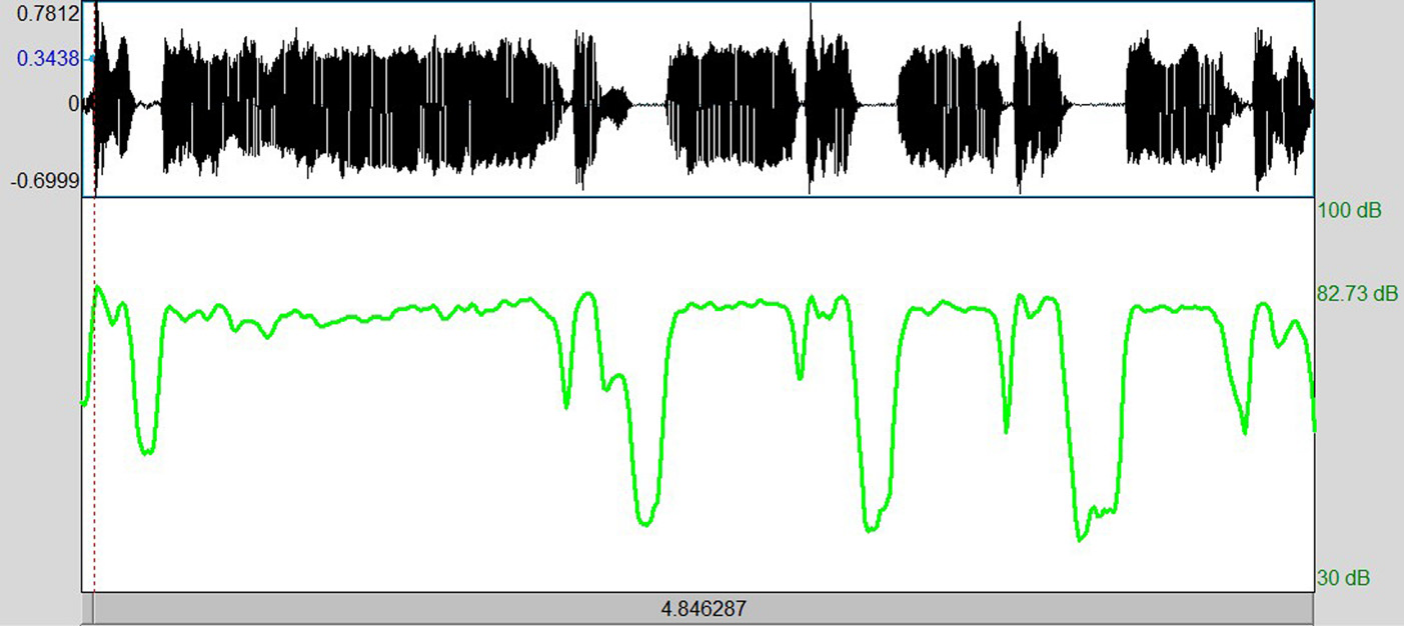
(Color online) Example of an intensity contour in Praat software, which is calculated on a consecutive expiration and inspiration with vocalization followed by a pause period.

We recently developed different approaches for cry segmentation (Abou-Abbas *et al.*, 2015b; Abou-Abbas *et al.*, 2015c). The general structure of the systems designed can be described with a block diagram as shown in Fig. 3.

We used machine-learning methods, such as HMMs and GMMs, which have been proven to work well in the acoustic domain. Different feature extraction techniques were employed and compared, reaching an accuracy rate of up to 91%. These methods were validated on our available signals through a tenfold cross-validation technique to perform training and testing operations of dissimilar sets of the total data.

However, there was a problem in precise boundary detection of the classified segments. This misdetection affects the accuracy rate of the overall system because the results of classification were compared to our manually segmented signals, which were labeled by trained colleagues. The exact cry and pause lengths between cries, although commonly overlooked by cry sound classification approaches, represent important parameters containing useful related information. For example, researchers in Várallyay (2006) used cry duration as an additional feature to detect the gender of the baby, and in Aucouturier *et al.* (2011), the duration of the expiration between contexts of cries, such as hunger, urinating, and sleepiness were investigated. Significant differences between durations have been observed. Lind *et al.* (1970) proved that cry durations are higher than usual in the cries of infants with Down's syndrome.

## V. CORPUS OF INFANTS' CRIES

Most of the subjects recruited for this study were newborns that were just a few days old. The size of our database and the diversity presented in our collected data distinguish our work from other aforementioned research studies:

• Signals collected in hospital environments at different times and in different situations, such as after birth, first bath, medical care, neonatal intensive care unit, and private or public maternity rooms with parents or visitors;
• Signals from crying babies with different contexts/causes such as pain, hunger, fear, and birth;
• Cry signals of babies with different pathological conditions such as normal infants and babies suffering from respiratory diseases, blood diseases, neurological diseases, or heart diseases.

In Table I, you can find a brief description of our cry database.

**TABLE I.** Description of our cry database.

Gender	Both male and female
Prematurity	Both preterm and full term
Babies ages	1–53 days old
Weight	0.98–5.2 Kg
APGAR (Appearance, Pulse, Grimace, Activity, Respiration) result	0–10 measured 2–3 times: once at birth, then 1, 5, and 10 min after the baby's birth
Gestational age	27 weeks and 2 days up to 41 weeks and 4 days
Origin	Canada, Haiti, Portugal, Syria, Lebanon, Algeria, Palestine, Bangladesh, Turkey
Race	Caucasian, Arabic, Asian, Latino, African, Native Hawaiian, Quebec
Reason for crying	Birth cry, hunger, dirty diaper, discomfort, needs to sleep, cold, pain, tummy troubles (colic, reflux, etc.)
Health condition	Healthy, heart diseases, respiratory diseases, neurological diseases, blood diseases, and other

The most accurate reason for crying was determined with the help of nurses and newborns' parents according to the situations that caused the infants to cry. The health condition was established by the doctor who examined the newborn and was based on different tests that had been performed in the hospital after the birth.

For recording purposes, an Olympus (Tokyo, Japan) handheld digital two-channel recorder was used and placed 10–30 cm from the newborn's mouth to be effective at a sampling frequency of 44.1 kHz and a sample resolution of 16 bits. There was no well-defined procedure during the acquisition of the cry sounds. Therefore, unwanted noises, cross talk, and clinical environment sounds were also recorded during the data collection process. For this reason, we consider our database a real corpus recorded in a real clinical environment. Our database in this work consists of a total of 507 waveforms of cry sounds interspersed by different unwanted acoustic activities. A summary of the database is given in Tables II and III, and it is the same database as used in our previous work (Abou-Abbas *et al.*, 2017) for comparison purposes.

**TABLE II.** Corpus statistics.

			Number of babies	Number of signals
Female	Full term	Healthy	56	141
		Pathological	34	94
	Preterm	Healthy	20	23
		Pathological	17	49
Male	Full term	Healthy	4	11
		Pathological	54	146
	Preterm	Healthy	5	11
		Pathological	13	32
Total			203	507

**TABLE III.** Data used for training and testing corpuses.

Classes	Time (sec)	Average time for training corpus/sec	Average time for testing corpus/sec
Expiration	21 414	19 348	2066
Inspiration	2154.8	1930	224.8
Background	5683.4	5228.1	455.3

To divide the dataset into training and testing corpuses, the tenfold cross-validation technique was applied.

## VI. MANUAL SEGMENTATION OF THE TRAINING AND TESTING DATA

**FIG. 4.**
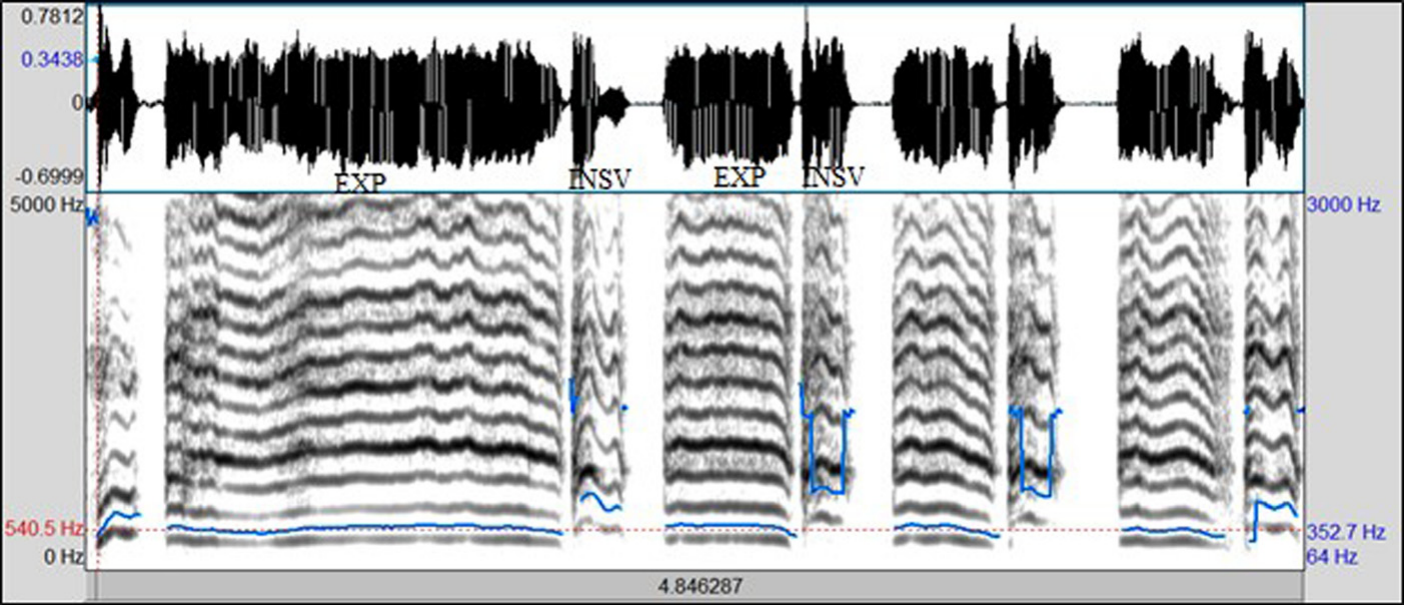
(Color online) Example of the fundamental frequency contour in Praat software, which is calculated on a consecutive expiration and inspiration with vocalization followed by a pause period.

**FIG. 5.**
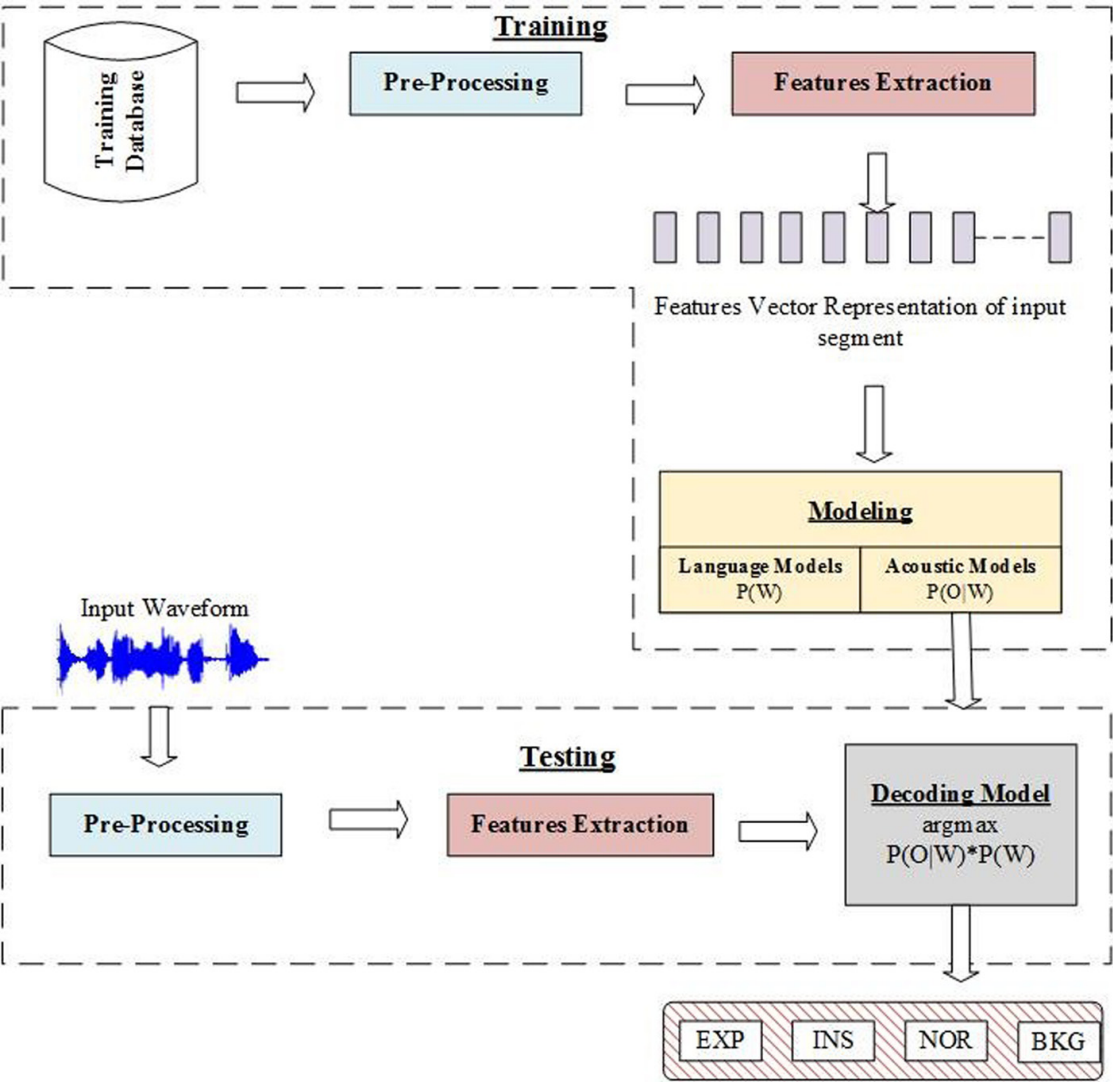
(Color online) Block diagram of a supervised cry segmentation system representing its training and testing stages.

**FIG. 6.**
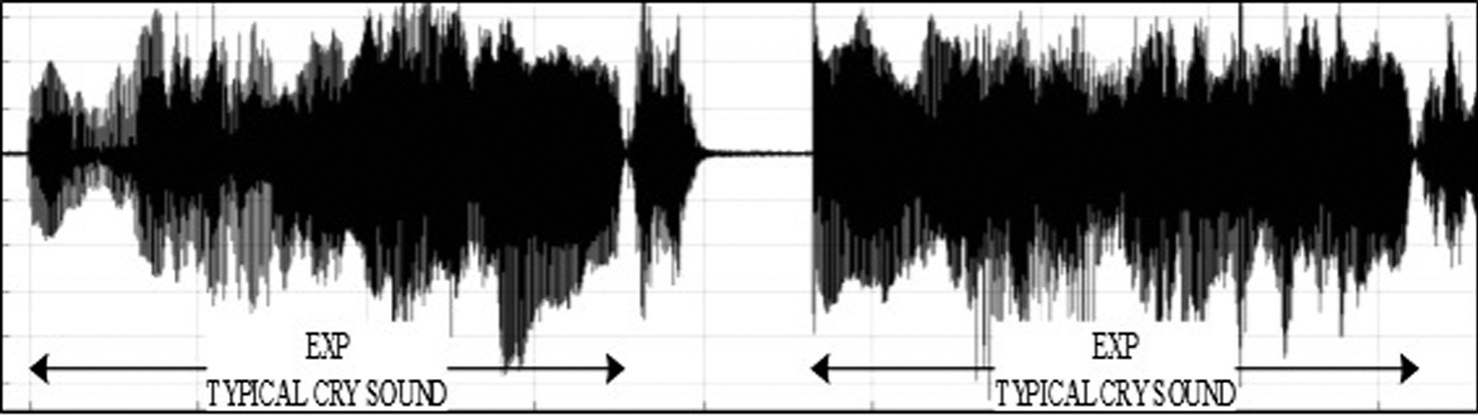
A waveform of the first type of expiration called typical cry sound (EXP) generated by a healthy newborn.

**FIG. 7.**
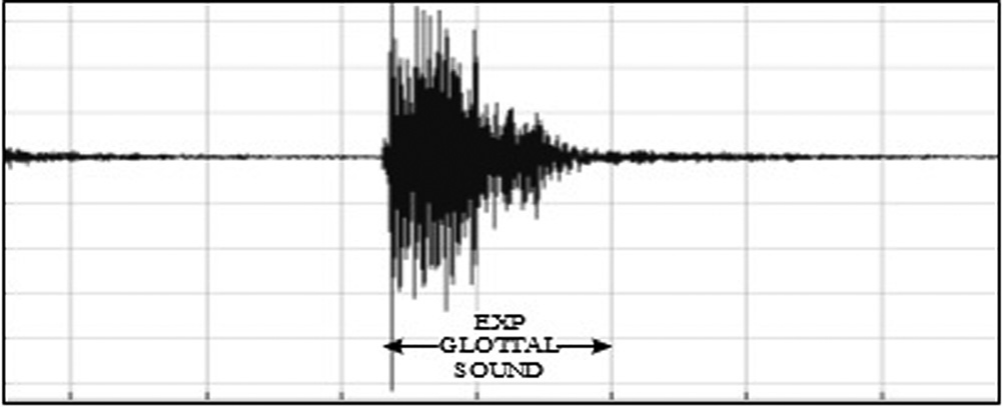
A waveform of the second type of expiration representing a cough sound during cry.

**FIG. 8.**
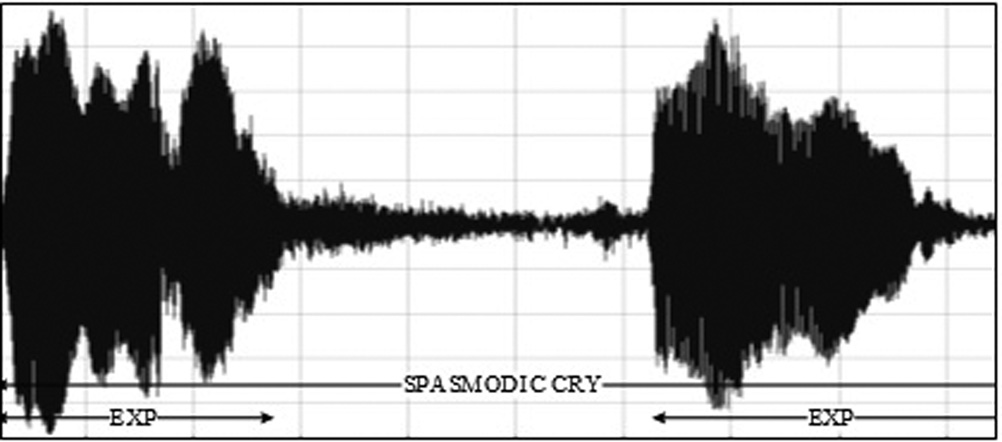
A waveform of a third type of expiration called spasmodic cry.

**FIG. 9.**
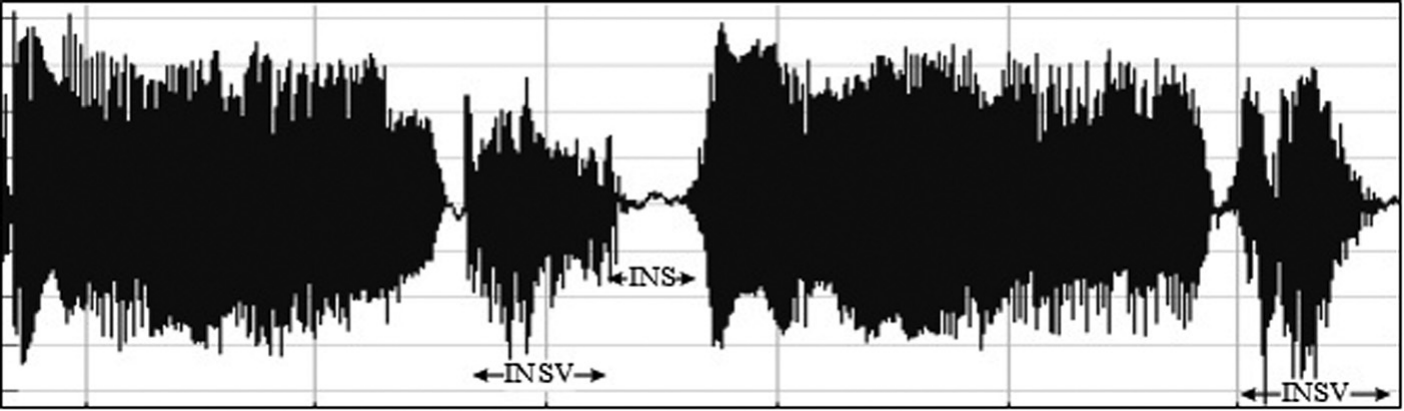
Waveform representing the difference between the INSV and INS labels.

**FIG. 10.**
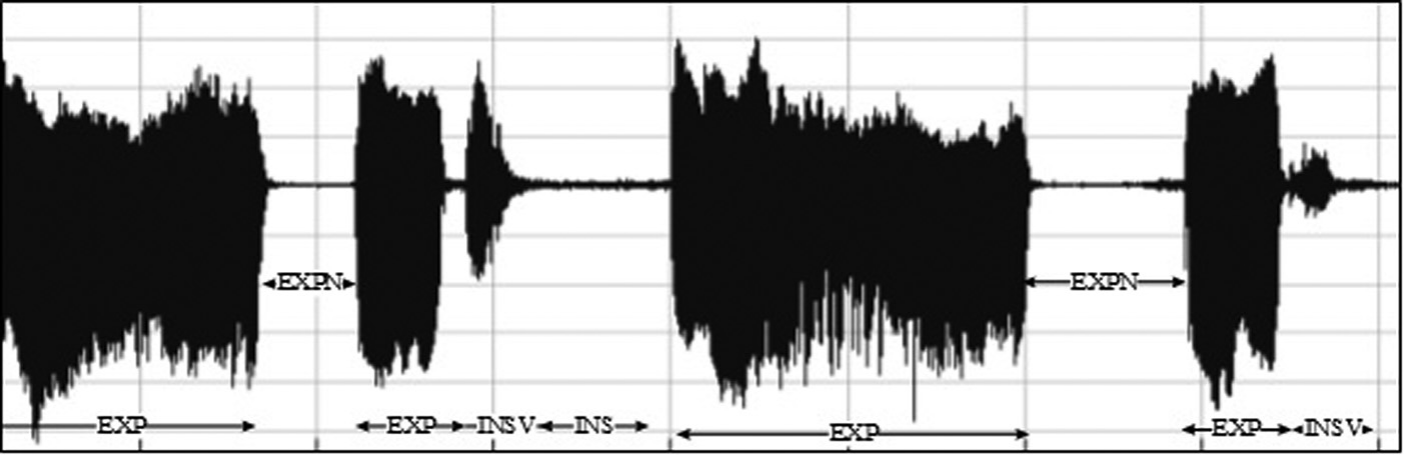
Babbling period labeled EXP2 or expiration generated after or before a cry.

Using the WaveSurfer software, we manually annotated recordings of our corpus (Sjölander and Beskow, 2000). Annotations identified the start and end points of each vocalization. The boundaries of each annotation were fixed by the point where the sound cannot be heard anymore. A newborn cry can comprise typical cry sounds, glottal sounds, hiccups, short pause segments between cries, and faint cries. The labels were chosen according to the different types of acoustic activities available in our corpora. The labels were defined as follows:

• EXP, which represents the total vocalization occurring during one expiration of a cry, as well as the sound between two inspirations (Lewis, 2007). It is composed of voiced or/and unvoiced segments. It is represented by three types: a typical cry sound (see Fig. 4), a glottal sound, i.e., a cough sound (see Fig. 5), or a spasmodic sound (see Fig. 6). We can easily distinguish typical expiration sounds from other expiration sounds by comparing their durations.
• INSV with vocalization, also called hiccups. It is the total vocalization occurring during one inspiration, and it usually follows a long EXP and is followed by a short pause segment. We noticed the presence of INSV in sick babies' cries more than those of healthy babies (see Fig. 7).
• Silent inspiration (INS), also called a short pause segment. It commonly happens between one INSV and the next EXP or directly after one EXP to be followed by another EXP (see Figs. 5 and 8).
• Expiration with non-vocalization (EXPN), also called a faint cry. It happens commonly after a long cry and always between two EXPs or EXP and INSV (see Fig. 8).
• EXP2 and INS2 represent components of vocalization during expiration and inspiration, respectively, and are produced by infants during babbling, cooing, etc. We could easily distinguish them from the EXP and INSV of the cry by their short durations (see Fig. 9).
• Speech (SP) of the medical staff or the parents around the baby. In general, speech signals have a low pitch range of 100–300 Hz (see Fig. 10).
• BIP is the sound of medical machines around the baby. It is characterized by a constant fundamental frequency.
• NOR or noises represent different types of sounds that can be either outside the cry or during the cry.
• BKG represents the background silence between cries, and it is a kind of a very low level noise (a low white noise).

## VII. PROPOSED APPROACH

In this paper, we developed a method for improving the accuracy rate of our previous systems based on a statistical approach via a post-processing step based on both temporal and frequency-domain features.

A post-processing stage was added for two reasons: (i) to minimize switching errors between the two predefined classes EXP and INSV, and (ii) to adjust the start and end points of each utterance.

Our method aimed to obtain more reasonable and accurate segmentation results by automatically applying additional steps to correctly and precisely classify cry components. Experiments were conducted to evaluate the utility of the post-processing stage and the use of additional temporal and frequency-domain information as acoustic features. The obtained results show that the use of our proposed approach for automatic segmentation was able to provide a major contribution to the performance of a cry segmentation system.

Our system uses a cascaded architecture to create an efficient cry segmentation system with a low false positive rate (FPR) while keeping the true positive rate (TPR) as high as possible.

In any acoustic recognition problem addressed in the literature, the following three concepts of signal processing were applied:

(1) Segmentation: First, a detector is used to segment the audio signal.
(2) Features extraction: The input signal is first converted into a series of acoustic vectors, which are then be forwarded to the recognition phase.
(3) Recognition: Features extracted are used to create a model to provide information about the active segments and take the best decision defined in the corresponding application.

The problem that arises from applying this traditional methodology concerns the adjustment of a static threshold, which should depend on the environmental conditions.

**FIG. 11.**
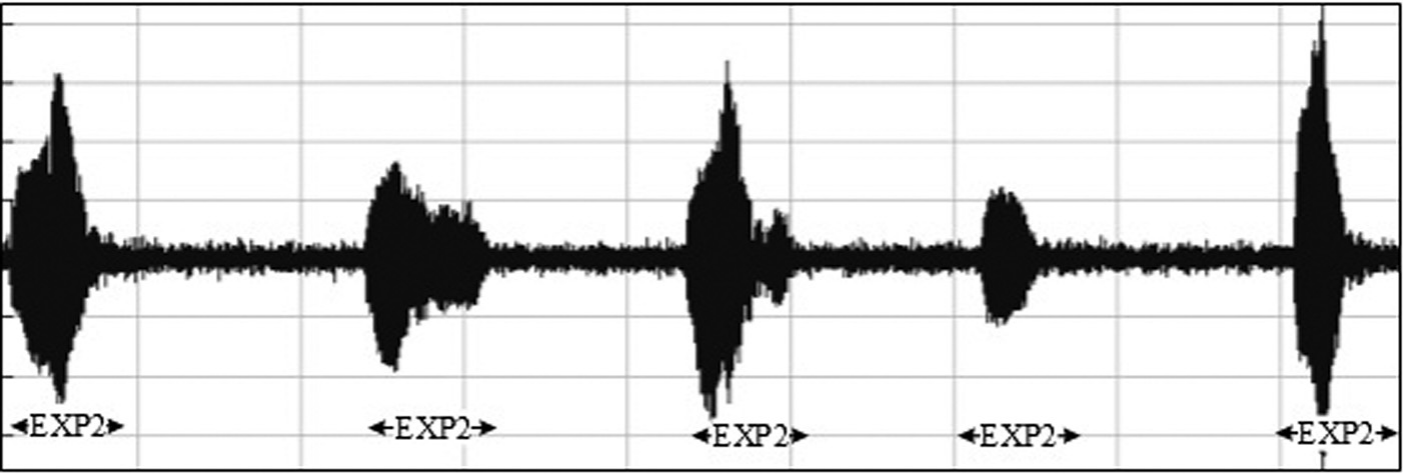
A cycle of a whole cry that contains EXP, INSV, INS, and EXPN.

Unlike this traditional methodology, we relied on frame-by-frame results obtained from standard classifiers to achieve a better segmentation by finding the exact boundaries of useful acoustic components. A number of features have been extracted, including MFCC, energy, intensity, ZCR, and fundamental frequency. We divided the proposed segmentation approach into two separate blocks. A block diagram of the main algorithm is depicted in Fig. 11.

In the first block, we chose HMM and GMM classifiers to model and classify each label (EXP/INSV/BKG and NOR, which represents all other acoustic activities, including noise). In the second block, a post-processing step followed the classification task. In this section, we give a general overview of the cry segmentation system and describe its different elements.

Our goal in this work was to automatically detect cries, expiration, and audible inspiration segments for a given recorded signal containing different acoustical activities such as crying, speech, silence, and different types of noises from common hospital environments.

### A. Pre-processing stage

**FIG. 12.**
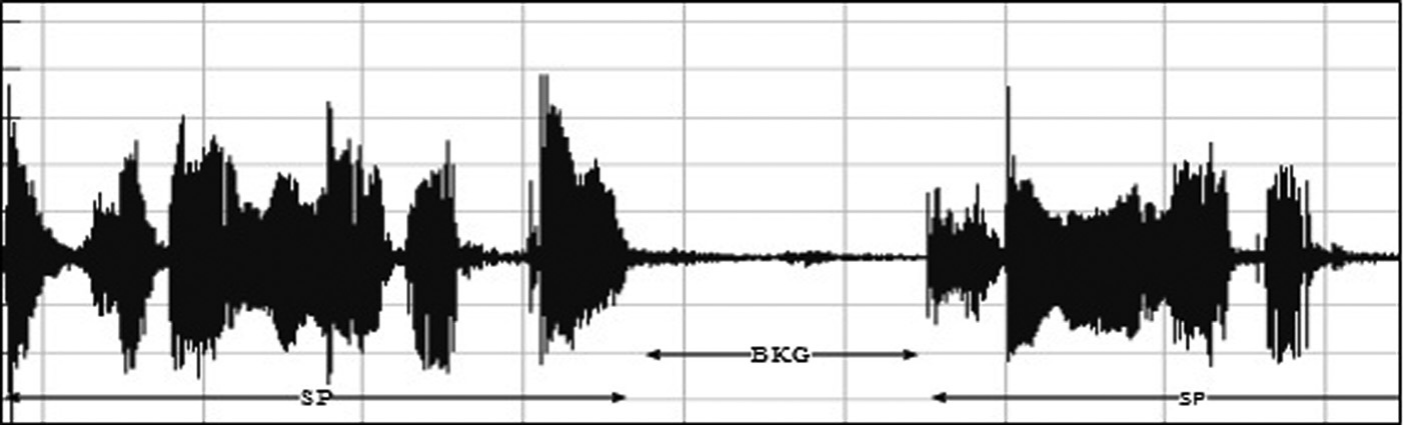
A waveform representing a human speech during a cry signal.

It is essential to have a pre-process step as the first step of any audio analysis system. The block diagram of our designed pre-processing stage is depicted in Fig. 12 and contains four modules:

(1) The input audio signal was converted to mono by calculating the average of both channels' audio files.
(2) A high-pass filter was applied to emphasize the signal. The most common filter is 1 – 0.97z^−1^.
(3) A framing module converted the continuous audio signal into overlapped frames based on frame size and overlap percentage.
(4) A hamming window was used to avoid the aliasing effect.

### B. Feature extraction procedure

A wide range of features can be extracted from the cry signals, but they do not necessarily correlate with a newborn's health condition. The selected feature set used in this work consisted of two parts: 39 features were used in the first classification stage (explained in Sec. VII A) to provide initial results, and another 5 features [ZCR, intensity, and fundamental frequency (minimum, maximum, and mean)] were used in the post-processing stage to give the final results.

The time/frequency-domain features that we employed are listed in Table IV.

**TABLE IV.** Set of features employed in the proposed method.

Domain	Features
Time	Zero crossing rate (ZCR)
	Sound intensity level (I)
Frequency	Min *f*0
	Max *f*0
	Mean *f*0
Time**-**frequency	FFT-MFCC
	EMD-MFCC

### C. Supervised cry segmentation system-initial classification

A supervised cry segmentation system is a chain of complex processing units that aims to locate cry sounds in audio signals collected in noisy clinical environments and identify its type as expiration, inspiration, background, or other classes based on trained models for all available classes in the training phase. In the testing phase, the probability that a segment belongs to each one of the classes is calculated using the Viterbi algorithm, and a decision is taken to produce a label for the segment.

In the works conducted previously (Abou-Abbas *et al.*, 2017; Abou-Abbas *et al.*, 2015b; Abou-Abbas *et al.*, 2015c), studies were carried out to compare the accuracy rate of different feature vectors and classifiers. It has been shown that all of the proposed approaches mentioned in Table V allow for reliable discrimination of clean expiration from any other sounds.

**TABLE V.** Set of experiments yielded using different decomposition techniques, different features extraction, and different classification methods.

Decomposition technique	Features extraction	Classification method
FFT	FFT-MFCC	GMM
FFT	FFT-MFCC	Four-states HMM
FFT	FFT-MFCC	Five-states HMM
EMD	EMD-MFCC	GMM
EMD	EMD-MFCC	Four-states HMM
EMD	EMD-MFCC	Five-states HMM

The first step of the new proposed cry segmentation scheme was to make the first classification decision based on a supervised cry segmentation system to discriminate between four different classes: EXP, INS, BKG, and other. We choose two previously designed systems to test alongside the new approach due to their robust performance. The window size used is 30 ms with an overlapping of 21 ms.

(1) Supervised cry segmentation system using a GMM classifier with 40 mixtures based on FFT-MFCC features.
(2) Supervised cry segmentation system designed using a 4-state HMM with 40 Gaussians classifier based on MFCC extracted from a combination of 3 IMFs: IMF3, IMF4, and IMF5 resulted from EMD.

Data used for training and testing the supervised cry segmentation systems are presented in Table II.

We also added delta and acceleration features to have a feature vector of 39 parameters per frame. We trained four different classes based on their available training data.

### D. Post-processing stage

**FIG. 13.**
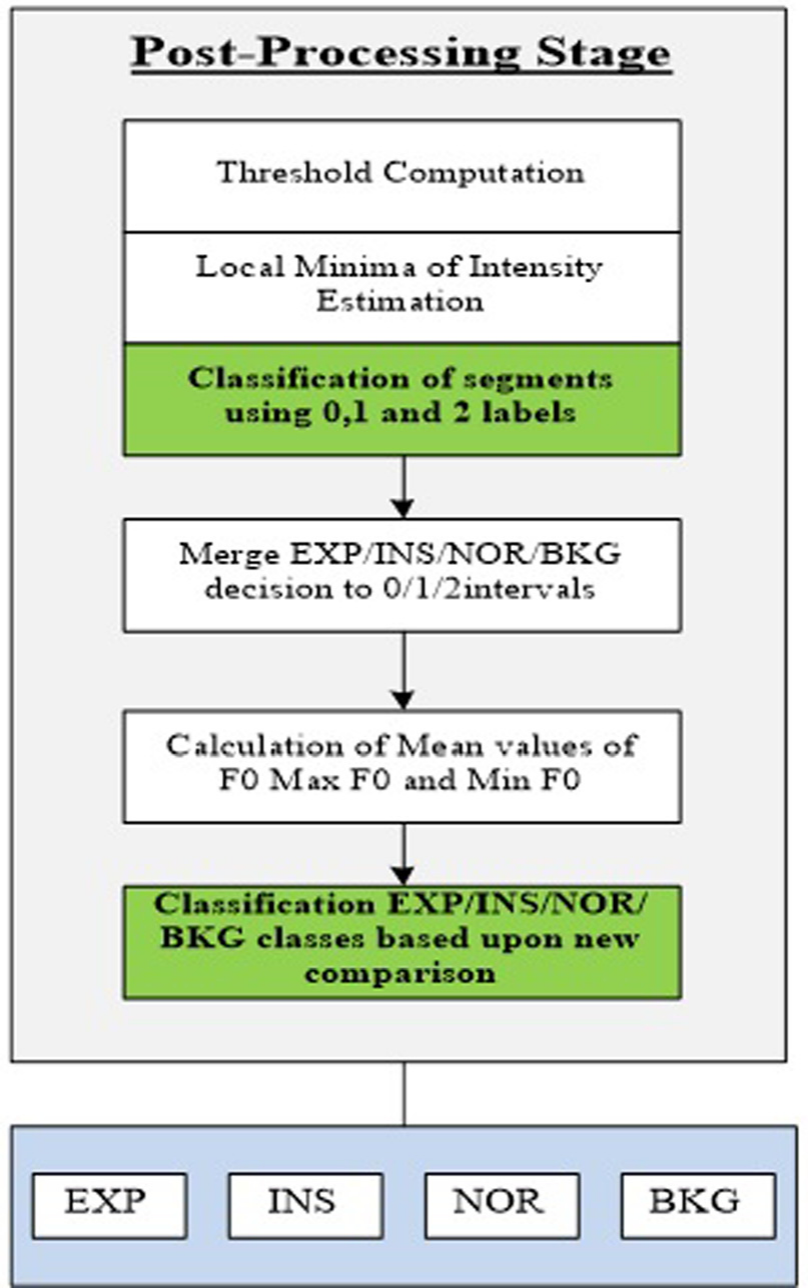
(Color online) Post-processing stage based on temporal features and frequency features.

To further improve the obtained classification results from the previous step, a post-processing stage depicted in Fig. 13 is proposed.

The main idea behind this stage was to refine the initially obtained results to reduce switching errors and adjust the boundaries of the expiratory and inspiratory phases. Then, to reline the classification results and make the final decision, we propose the second step based on temporal features and frequency features separately.

**FIG. 14.**
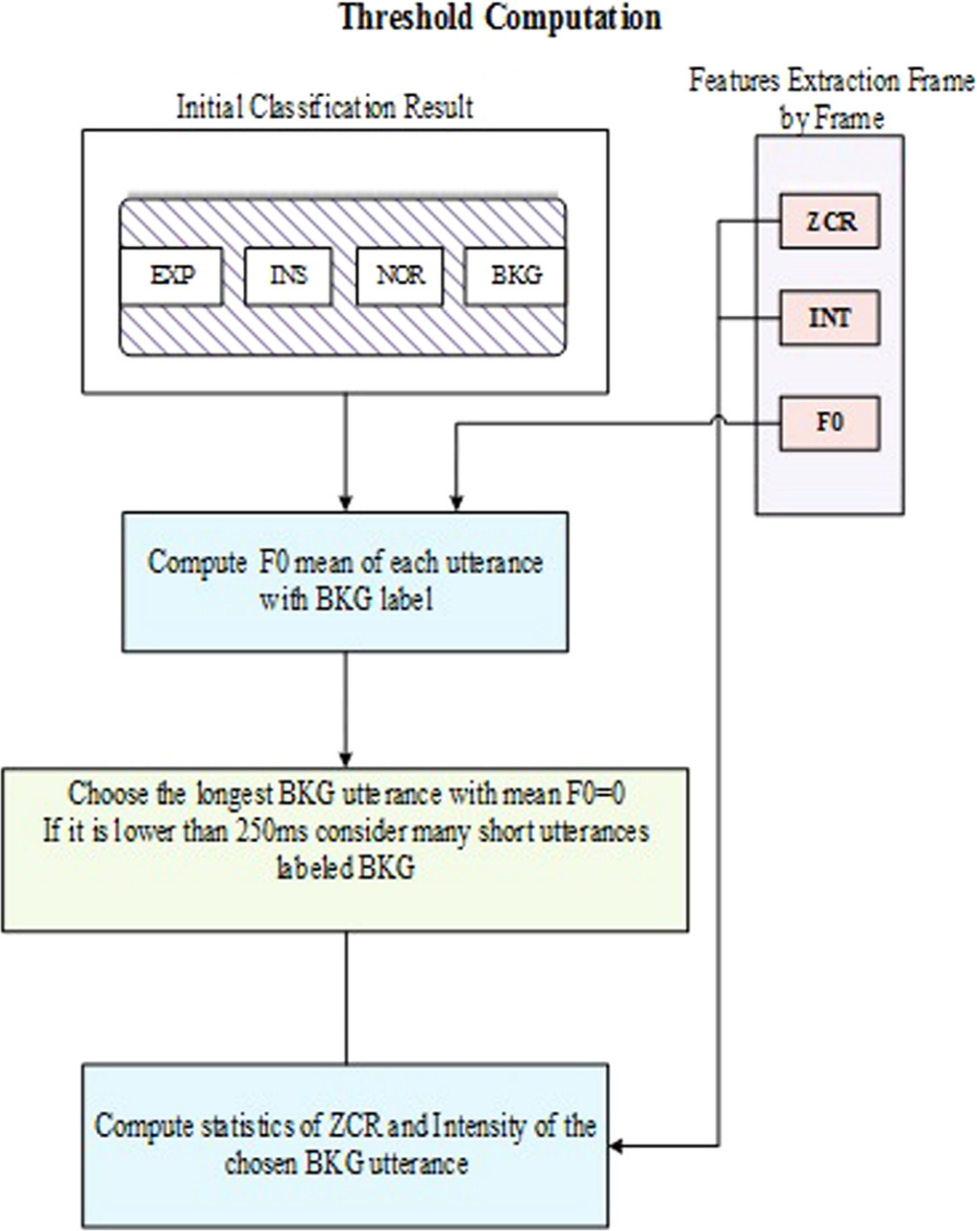
(Color online) Dynamic threshold computation module based on the initial classification results of EXP, INS, NOR, and BKG for the calculation of ZC_BKG and INT_BKG.

In particular, the following steps were taken in this stage:

(1) First, the results obtained from the initial classification step (in label files) were further taken as input to the post-processing phase.
(2) Different features were extracted for each frame from the corresponding input wave file such as ZCR, intensity, and fundamental frequency.
(3) Two thresholds were calculated: ZC_BKG and INT_BKG. It was based on the Rabiner and Sambur rules in the endpoint detection algorithm (Rabiner and Sambur, 1975). The threshold computation module explained in Fig. 14 is an essential step in estimating measures of background silence. After computing the intensity vector of the 250 ms of the silence called INT_sil, the intensity vector of the whole signal called INT_sig and the ZCR vector of the silence segment called ZCR_sil thresholds are then calculated according to the following equation:
  $$\begin{array}{c} \text{ZC\_BKG}=\left(\text{mean}\left(\text{ZCR\_sil}\right)+2\text{std}\left(\text{ZCR\_sil}\right)\right)\\ I_{\text{mean}}=\text{mean}(\text{INT\_sil});\\ I_{\max}=\text{max}(\text{INT\_sig});\\ I_{1}=0.03(I_{\max}-I_{\text{mean}})+I_{\text{mean}};\\ I_{2}=4I_{\text{mean}};\\ \text{ITL}=\text{min}(I_{1},I_{2});\\ \text{INT\_BKG}=5ITL. \end{array}$$
(4) Each frame was labeled as “0” and “1” based on the *F*0 results. If *F*0 value exists, the frame was indexed by “1,” and if *F*0 does not exist for the frame, it was indexed by “0.” Table VI shows an example of indexing frames.
(5) The localized points were predicted from the transition between 0 and 1 among consecutive segments. These frames were indexed by “2” to determine the transitions within the localized points. See columns 2 and 3 in Table VI.
(6) Then, all local minima positions of intensity were indexed separately from the localized points. See column 4 in Table VI.
(7) Two different indexes were gathered to create new intervals. The start and end of each interval were marked by the index “2.” See column 5 in Table VI.
(8) Calculate the mean values of the intensity and ZCR.
(9) Compare the intensity values and ZCR values with the thresholds (INT_BKG and ZC_BKG) and give new index labels of 0 and 1 to the intervals. See Table VII.
(10) Merge the decisions of the initial classification with the new indexes to create new improved intervals. See Table VIII.
(11) Calculate the mean of the intensity to create new intervals, and add *F*0 (mean, max, min) values for each new interval.
(12) Re-compare the mean values of the intensity and fundamental frequency statistics to make the final decision on utterances.

**TABLE VI.** Process of threshold computation—Steps 3–7.

Frame number	First labeling	Marking transition	Marking local minima	Combination of three columns indexes
1	1	x	2	2
2	1	x	x	1
3	1	x	x	1
4	1	x	x	1
5	1	2	x	2
6	0	x	x	0
7	0	x	2	2
8	0	x	x	0
9	0	2	x	2
10	1	x	x	1
11	1	x	2	2
12	1	x	x	1
13	1	2	2	2
14	0	x	x	0
15	0	x	x	0
16	0	x	x	2

**TABLE VII.** Process of threshold computation—Steps 8 and 9.

Intervals from frame *i* to frame *j*	Intensity	ZCR	New index
1–5	x	x	0
6,7	x	x	0
8,9	x	x	1
10,11	x	x	1
12,13	x	x	0
14–16	x	x	1

**TABLE VIII.** Process of threshold computation—Step 10.

Frame number	Initial classification results	New index	Combination
1	BKG	0	BKG0
2	BKG	0	BKG0
3	BKG	0	BKG0
4	BKG	0	BKG0
5	BKG	0	BKG0
6	EXP	0	EXP0
7	EXP	0	EXP0
8	EXP	1	EXP1
9	EXP	1	EXP1
10	INS	1	INS1
11	INS	1	INS1
12	INS	0	INS0
13	INS	0	INS0
14	NOR	1	NOR1
15	NOR	1	NOR1
16	NOR	1	NOR1

## VIII. RESULTS AND DISCUSSION

The proposed method in this work was tested on a set of 507 cry signals collected from 203 babies. The performance was estimated at the frame level by comparing each classified frame to the reference frame (the results of manual segmentation performed by experts). Because the main advantage of this work is robustness, the systems were trained and tested using recorded cry signals under varying conditions, as mentioned in Sec. III.

Four different statistical measures were derived for each class: true positive (TP), false positive (FP), true negative (TN), and false negative (FN). TP is the number of correctly detected sounds; FP is all sounds detected erroneously; TN represents the number of correctly rejected sounds; FN is all missed sounds.

The performance of the proposed approach was evaluated using metrics such as TPR, FPR, false negative rate (FNR), and accuracy.

The first set of experiments was conducted with two supervised segmentation systems using GMM and HMM classifiers, respectively. Tenfold cross-validation was used to evaluate the performance of the system in a manner such that onefold was reserved for validation while the remaining ninefolds constitute the training set. This procedure was performed five times, and the average accuracies depicted in Table IX were obtained.

**TABLE IX.** Results of different experiments. Bold entries represent the best averages obtained for detecting expiration and inspiration phases.

	EXP			INS			BKG			
%	TPR	FPR	FNR	TPR	FPR	FNR	TPR	FPR	FNR	Overall accuracy
FFT-GMM	93.03	18.52	6.97	84.56	29.65	15.44	95.63	10.36	3.37	91.01
EMD-HMM	88.32	23.6	11.68	90.35	27.74	9.65	92.85	19.6	7.15	88.94
FFT-GMM + post-processing	**95.6**	**10.32**	4.4	89.52	20.46	10.48	97.26	3.23	2.74	**94.29**
EMD-HMM + post-processing	93.7	15.08	6.3	**92.47**	**18.21**	7.53	95.07	7.84	4.93	92.16

In our experiments, the GMM- and HMM-based classifiers were trained, respectively, by FFT-MFCC and MFCC features extracted from the combination of three IMFs, IMF3, IMF4, and IMF5 resulted from EMD.

The second set of experiments, which was designed to evaluate the post-processing stage, was further conducted using results obtained from the first set of experiments. The results obtained before and after the post-processing stage are shown in Table IX.

**FIG. 15.**
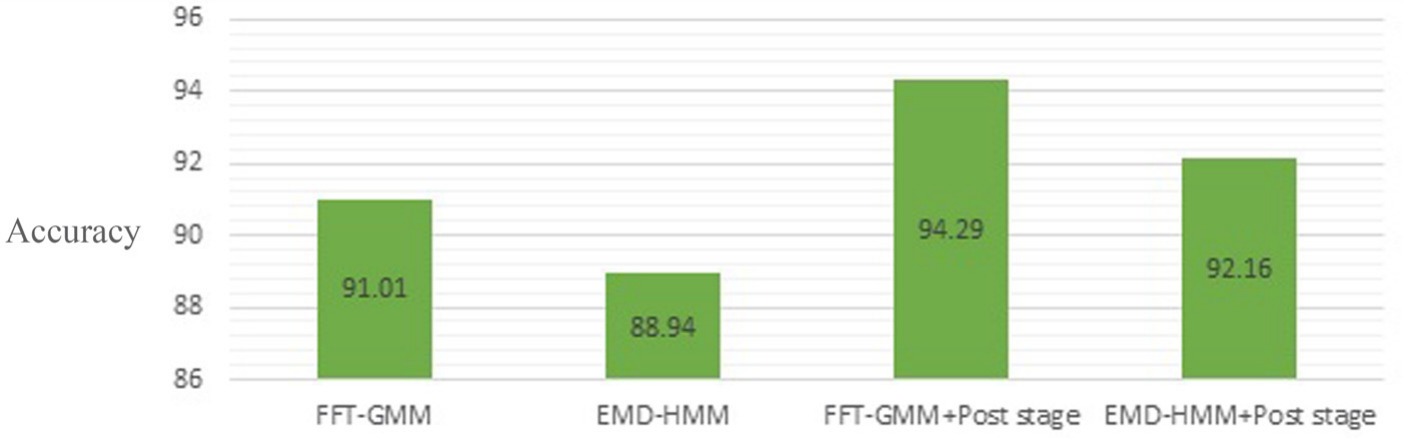
(Color online) Comparison of the overall accuracy rate obtained by FFT-GMM, EMD-HMM, FFT-GMM with the post stage, EMD-HMM with the post stage.

Table IX shows the average TPR, FPR, FNR values of the proposed approach and the previous designed systems. Based on the results, the overall accuracies of FFT-GMM- and EMD-HMM-based systems improved by approximately 3.18% and 3.22%, respectively, by applying the post-processing stage (Fig. 15).

**FIG. 16.**
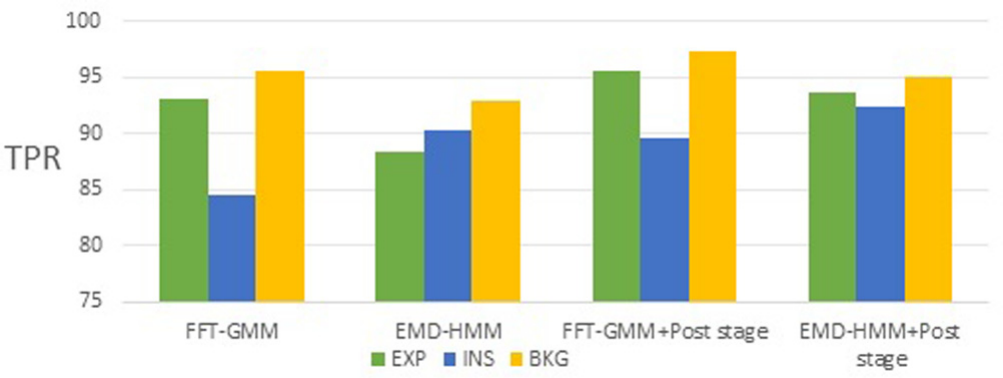
(Color online) Comparison of TPR among three different classification systems proposed for classes EXP, INS, and BKG.

The proposed method achieved a high average TPR of approximately 95.6% for the EXP class and 92.47% for the INS class by using GMM and HMM classifiers, respectively. Figure 16 shows a comparison of the TPR results obtained for the four experiments mentioned in Table IX.

**FIG. 17.**
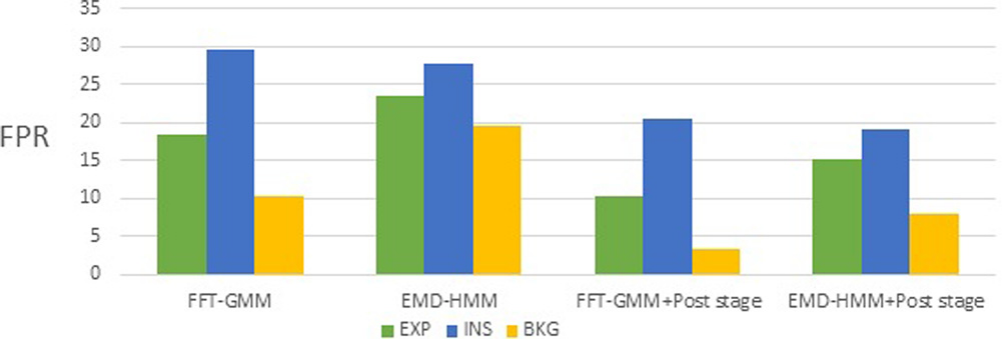
(Color online) Comparison of FPR among the three different classification systems proposed for classes EXP, INS, and BKG.

The FPR was considered the most important error rate in our case because it is very important not to add misleading segments as input to a cry classification system. As we can see from Table IX and Fig. 17, the post-processing stage reduced the FPR for the EXP and INS classes by 8.2% and 11.44%, respectively.

Moreover, an average FPR rate of approximately 10.32% was obtained for the EXP class using the GMM-based classifier and 18.21% for the INS class with the HMM-based classifier.

From the experiments, it can be seen that the proposed method achieved an average performance of 94.29%.

Based on the results, we can see that an improvement of 3.18% in the average classification rates was achieved by adding the post-processing stage, which was quite significant.

## IX. CONCLUSION

Cry segmentation is the process of segmenting a cry signal recorded in a real clinical environment into homogenous regions. When used in conjunction with an NCDS, an automatic cry segmentation system is able to detect useful cry units with vocalizations from non-cry audio regions (such as speech, machine sounds, and other types of noises) and significantly improve the intelligibility of the NCDS.

Our main goal was to precisely locate important audible cry component boundaries of continuous recordings collected in a very noisy environment as a step toward building a cry database that contributes to developing different cry-based applications such as the following:

• Distinction between diverse types of cries: birth cry, pain cry, normal cry, pleasure cry, etc.;
• Classification between healthy and deaf infants;
• Distinction between healthy infants and infants with asphyxia;
• Discrimination of babies with cleft palates with palate plates from babies with cleft palates but without palate plates;
• Classification of pathology from cries: asphyxia, meningitis;
• Recognition of newborns' native language from cry melodies;

Having a large enough database that is clean, well-recorded and segmented for training steps is a critical parameter for each of the mentioned applications.

The main goal of the proposed work was to design an automatic cry segmentation system that can be incorporated in the early stage of our NCDS system or any other cry classifier system.

In this work, expiratory and inspiratory cries with vocalization were detected from audio signals containing different acoustic activities other than cries. A post-processing stage was combined with a supervised cry segmentation system that has already been designed in our previous work. The proposed approach achieved better performance in terms of accuracy rate, TPR, and FPR.

Future work will include a combination of GMM- and HMM-based classifiers because the GMM-based classifier gave fewer false-positive alarms for the EXP class, while the HMM-based classifier gave fewer false-positive alarms for the INS class. Moreover, the final boundaries of EXP and INS phases could be used to extract temporal features such as the duration of the expiratory and inspiratory phases, duration of pauses between cries, and onset of crying, which may result in a more accurate pathology classification system.
